# Hypertonic Saline Versus Other Intracranial-Pressure-Lowering Agents for Patients with Acute Traumatic Brain Injury: A Systematic Review and Meta-analysis

**DOI:** 10.1007/s12028-023-01771-9

**Published:** 2023-06-28

**Authors:** Keeley Bernhardt, William McClune, Matthew J. Rowland, Akshay Shah

**Affiliations:** 1 University of Oxford Medical School, Oxford, UK; 2 Wellcome Wolfson Institute for Experimental Medicine, Queen’s University, Belfast, UK; 3 Cardiovascular, Renal, and Metabolism Group, Novartis, London, UK; 4 Nuffield Department of Clinical Neurosciences, University of Oxford, Oxford, UK

**Keywords:** Traumatic brain injury, Intracranial pressure, Osmotherapy, Hypertonic saline

## Abstract

Acute traumatic brain injury (TBI) is a major cause of mortality and disability worldwide. Intracranial pressure (ICP)-lowering is a critical management priority in patients with moderate to severe acute TBI. We aimed to evaluate the clinical efficacy and safety of hypertonic saline (HTS) versus other ICP-lowering agents in patients with TBI. We conducted a systematic search from 2000 onward for randomized controlled trials (RCTs) comparing HTS vs. other ICP-lowering agents in patients with TBI of all ages. The primary outcome was the Glasgow Outcome Scale (GOS) score at 6 months (PROSPERO CRD42022324370). Ten RCTs (760 patients) were included. Six RCTs were included in the quantitative analysis. There was no evidence of an effect of HTS on the GOS score (favorable vs. unfavorable) compared with other agents (risk ratio [RR] 0.82, 95% confidence interval [CI] 0.48–1.40; *n* = 406; 2 RCTs). There was no evidence of an effect of HTS on all-cause mortality (RR 0.96, 95% CI 0.60–1.55; *n* = 486; 5 RCTs) or total length of stay (RR 2.36, 95% CI − 0.53 to 5.25; *n* = 89; 3 RCTs). HTS was associated with adverse hypernatremia compared with other agents (RR 2.13, 95% CI 1.09–4.17; *n* = 386; 2 RCTs). The point estimate favored a reduction in uncontrolled ICP with HTS, but this was not statistically significant (RR 0.52, 95% CI 0.26–1.04; *n* = 423; 3 RCTs). Most included RCTs were at unclear or high risk of bias because of lack of blinding, incomplete outcome data, and selective reporting. We found no evidence of an effect of HTS on clinically important outcomes and that HTS is associated with adverse hypernatremia. The included evidence was of low to very low certainty, but ongoing RCTs may help to the reduce this uncertainty. In addition, heterogeneity in GOS score reporting reflects the need for a standardized TBI core outcome set.

**Supplementary Information:**

The online version contains supplementary material available at 10.1007/s12028-023-01771-9.

## Introduction

Acute traumatic brain injury (TBI) is a major cause of mortality and disability worldwide [1]. In the United Kingdom, TBI is the most common cause of death in patients under 40 years of age [2]. Raised intracranial pressure (ICP) secondary to TBI increases the risk of brain herniation and is associated with poorer clinical outcomes [3]. Thus, lowering ICP is a critical management priority in patients with moderate to severe acute TBI.

Hyperosmolar therapies, such as hypertonic saline (HTS) and mannitol, are in routine clinical use for lowering ICP in TBI. Historically, both agents were thought to produce an ICP-lowering effect by drawing interstitial fluid within edematous brain tissue intravascularly. More recently, their mechanism of action is increasingly understood to involve complex alterations in blood viscosity and microcirculatory changes resulting in pial arteriolar constriction, decreased cerebral blood volume, and reduced ICP [4, 5]. Despite increasing popularity of HTS in this setting and positive results from previous studies suggesting potential clinical benefits, the most recent Brain Trauma Foundation guidelines (2016) state that there was “insufficient evidence available from comparative studies to support a formal recommendation” for its use [6–8]. Severe hypernatremia has been noted as a potential adverse effect associated with HTS use [9]. Moreover, a recent Cochrane review concluded that there was weak evidence to suggest HTS has no effect on long-term neurological outcome compared with mannitol, although this review was released prior to publication of the largest randomized trial investigating HTS infusion in patients with acute TBI (the continous hyperosmolar therapy for traumatic brain-injured patients (COBI) trial) [10, 11]. The COBI trial included 370 adults with moderate to severe TBI and found no evidence of an effect of a continuous HTS infusion compared with standard care on long-term neurological function.

Therefore, it remains unclear whether HTS offers any clinical benefit over other ICP-lowering methods in terms of long-term functional outcome, all-cause mortality, ICP control, and adverse effects. This review seeks a definitive answer to this question to guide clinical practice and inform future research.

## Methods

This report was prepared according to the Preferred Reporting Items for Systematic Reviews and Meta-Analyses (PRISMA) reporting guideline [12]. Our review protocol was prospectively registered on PROSPERO (CRD4202234370).

### Eligibility Criteria

We included randomized controlled trials (RCTs) comparing the effect of HTS bolus(es) or infusion versus other ICP-lowering agents on clinical outcomes of interest in patients of all ages receiving critical care for acute TBI. Because HTS was licensed as a hyperosmolar agent for lowering ICP in 2004, studies were selected from 2000 onward to ensure that results are reflective of current clinical practice. Nonhuman studies, conference abstracts, and those published in languages other than English were excluded.

**Fig. 1:**
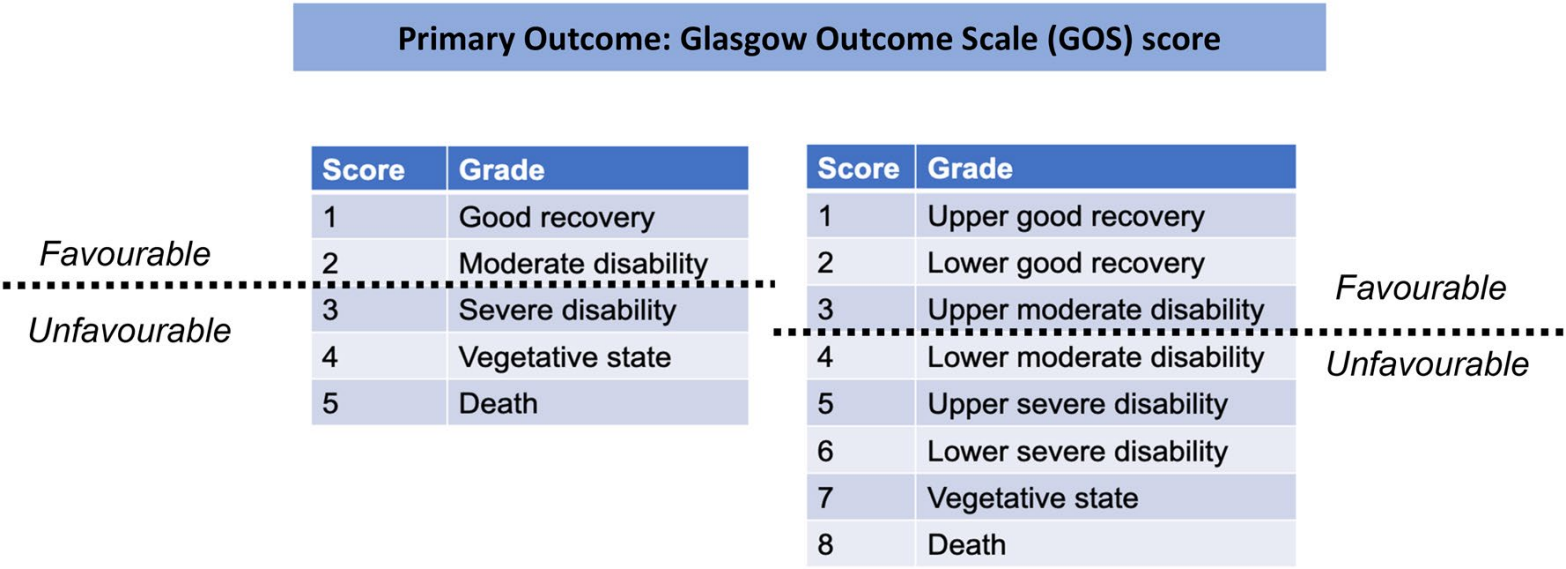
Glasgow outcome scale (GOS) score dichotomization into “favorable” and “unfavorable” outcomes

Our primary outcome was “favorable” Glasgow Outcome Scale (GOS) score at 6 months [13] (Fig. 1). A full description of GOS score criteria is provided in the Supplementary Material. Secondary outcomes were all-cause mortality, changes in ICP, proportion of patients with uncontrolled ICP, length of stay (hospital and/or intensive care unit [ICU]), and adverse events, including pulmonary edema and rebound phenomenon.

### Search Strategy

MEDLINE, Cochrane CENTRAL (Cochrane Central Register of Controlled Trials), Embase, ISI (Institute for Scientific Information) Web of Science, Scopus, and clinical trial registries (ClinicalTrials.gov, World Health Organization International Trials Registry, Chinese Clinical Trials Registry) were initially searched on April 10, 2022, according to a predefined search strategy for each database. This search was repeated on November 19, 2022. The search strategies were developed in collaboration with an experienced librarian from Bodleian Libraries, University of Oxford. Reference lists of identified trials were searched for further relevant literature, and individual study authors were contacted to request additional data if necessary. Key search terms included “hypertonic saline,” “traumatic brain injury,” and “intracranial pressure.” Individual search strategies can be found in the Supplemental Material.

### Study Selection and Data Extraction

Titles, abstracts, and full texts of identified studies were screened in duplicate by two independent authors (KB, WM) against prespecified inclusion and exclusion criteria (see Eligibility criteria section). Any discrepancies in the screening process were discussed until consensus was reached, and in the event of a disagreement, a third author (AS) was assigned to adjudicate. Study authors were contacted if additional data were required for inclusion in the quantitative analysis. Data were extracted in duplicate by two independent authors using a pre-piloted spreadsheet.

### Risk of Bias Assessment

The Cochrane Collaboration’s domain-based Risk of Bias 1 tool was used to assess risk of bias for each included study. Any discrepancies were discussed until consensus was reached. A third assessor was approached to adjudicate if consensus was not possible. Adjudication was only required on one occasion.

### Data Synthesis

Data were entered into the Cochrane Collaboration’s systematic review software (RevMan 5, 2011) Heterogeneity between studies was assessed with the use of *I*^2^ [14]. Data were synthesized to obtain pooled estimates of relative risks (95% confidence interval [CI]) or mean difference (95% CI) as appropriate using a random-effects model for primary and secondary outcomes. Owing to variations in reporting of GOS scores between studies, the primary outcome (GOS score at 6 months) was dichotomized into “favorable” or “unfavorable” functional outcome (Fig. 1). This review outcome was reported as a pooled risk ratio (RR) with a corresponding 95% CI. Forest plots were produced for each outcome of interest. Where possible, continuous variables were reported as weighted mean or standardized mean difference as appropriate.

Where data could not be pooled, narrative syntheses were performed. Subgroup analyses focusing on administration factors, age group, and TBI severity were prespecified to determine whether these factors affect outcomes of interest. Moreover, a sensitivity analysis was planned to investigate the influence of high risk of bias studies. However, few studies were identified for inclusion, and the majority of these consisted of small sample sizes. This precluded our ability to perform further meaningful subgroup or sensitivity analyses using currently available data.

### Certainty of Evidence

The Grading of Recommendations Assessment, Development, and Evaluation (GRADE) approach was used to assess the overall certainty of the evidence [15].

## Results

**Fig. 2:**
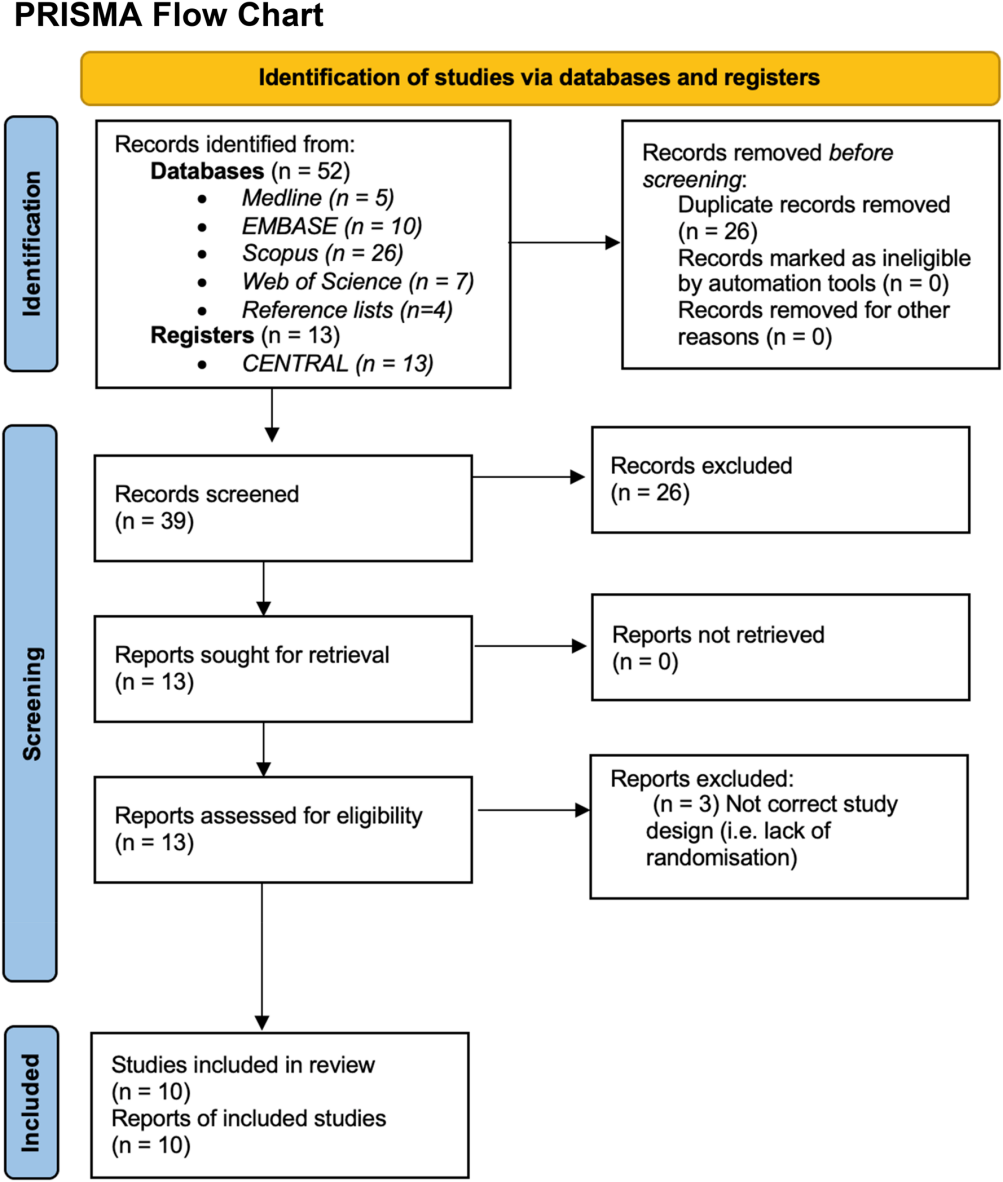
Preferred Reporting Items for Systematic Reviews (PRISMA) flow chart showing studies identified, screened, and included in the systematic review

Of 65 studies identified, 13 underwent full-text screening after title and abstract screening (Fig. 2). Three studies were excluded after full-text screening because of incorrect study design. Of the ten remaining studies, six were included in the meta-analysis and three were included in narrative syntheses. One ongoing multicenter RCT (Sugar or Salt) was identified (ISRCTN16075091).

### Description of Included Studies

Details of the included trials are shown in Table 1. The ten trials comprised a total of 760 patients receiving critical care for brain injury in the countries France, India, Iran, Germany, Egypt, and Israel. There were only three multicenter trials. Two trials included patients with spontaneous intracerebral or subarachnoid hemorrhage in addition to patients with acute TBI. Because TBI subgroup data were unavailable for both trials, these were included in narrative syntheses and omitted from the meta-analysis.

**Table 1:** Characteristics of included studies (*n* = 10)

Study author	Methods	Participants	Intervention	Comparator(s)	Primary outcome	Secondary outcome (s)
Cottenceau et al. [16]	Multicenter (*n* = 2), parallel RCT (France and Israel)	Patients 16 + years old with severe TBI requiring ICP monitoring and mechanical ventilation GCS 8 or below at time of admission *N* = 47	2-mL/kg dose 7**.**5% hypertonic saline bolus infused intravenously over 20 min	4-mL/kg dose 20% mannitol bolus infused intravenously over 20 min	Maximal ICP reduction and change in cerebral perfusion pressure from baseline after hyperosmolar therapy (study period of 120 min)	Neurological outcome at 6 months (GOS) All-cause mortality by 6 months Uncontrolled ICP, defined as average time ICP exceeded 20 mm Hg Hypernatremia
Francony et al. [17]	Single-center parallel RCT (France)	Adult patients 18 + years old with sustained elevation of ICP greater than 20 mm Hg for more than 10 min Included patients with TBI, stroke, and spontaneous hemorrhage Mechanically ventilated and in stable condition for > 2 h prior to study commencement according to predefined criteria *N* = 20	100 mL of 7.45% hypertonic saline bolus infused via CVC over 20 min	231 mL of 20% mannitol bolus infused via CVC over 20 min	ICP magnitude and change in cerebral perfusion pressure after hyperosmolar therapy during the 120-min monitoring period	Rebound phenomenon
Harutjunyan et al. [18]	Single-center parallel RCT (Germany)	Neurosurgical adult patients > 18 years old with severe neuronal damage and at risk of increased ICP Included patients with cerebral trauma, spontaneous intracerebral hemorrhage, or subarachnoid hemorrhage *N* = 32	Variable dose 7.2% hypertonic saline and hydroxyethyl starch 200/0.5 via CVC at variable rate until ICP < 15 mm Hg	Variable dose 15% mannitol via CVC at variable rate until ICP < 15 mm Hg	ICP reduction below 15 mm Hg (% maximal decrease in ICP from baseline)	All-cause mortality by 6 months Refractory ICP (> 15 mm Hg) requiring thiopentone administration
Hendoui et al. [19]	Multicenter (*n* = 3), 3-arm parallel RCT (Iran)	Adult (18–65 years old) patients with GCS 12 or below and evidence of brain edema on CT scan All patients had TBI (*N* = 33)	Intervention 1: 125-mL 5% hypertonic saline bolus dose infused over 1 h every 6 h for 3 days Intervention 2: 500-mL 5% hypertonic saline continuous infusion over 3 days	1-g/kg 20% mannitol bolus dose infused over 20 min 0.25–0.5 g/kg repeat dose every 6 h based on response to previous dose for 3 days	GCS reduction (used to infer reduction of brain edema) S100B biomarker measurement	Hypernatremia Mortality and 60-day survival Length of ICU and hospital stay
Jagannatha et al. [20]	Single-center parallel RCT (India)	Patients with severe TBI aged 15–70 years old (children and adults) within 24 h of injury (*N* = 38)	2.5-mL/kg bolus dose 3% hypertonic saline infused via CVC over 5 min; maximum of 3 doses given	2.5-mL/kg bolus dose 20% mannitol infused via CVC over 5 min; maximum of 3 doses given	Lowest ICP achieved for each bolus and time required to achieve ICP < 20 mm Hg	Duration of ICU and hospital stay In-hospital mortality GOS at 6 months Uncontrolled ICP, defined as sustained ICP > 20 mm Hg after maximum of 3 doses hyperosmolar therapy, requiring barbiturate administration Hypernatremia
Kumar et al. [21]	Single-center parallel RCT (India)	Children (1–16 years old) with severe TBI and pediatric GCS of 8 or less presenting within 24 h of trauma *N* = 30	2.5-mL/kg bolus dose 3% hypertonic saline infused via CVC over 5 min Second dose given if first bolus failed	2.5-mL/kg bolus dose 20% mannitol infused via CVC over 5 min Second dose given if first bolus failed	Mean reduction in ICP, defined as the difference between baseline ICP and lowest ICP after completion of bolus for each dose	GOS score at 6 months (modified for children) Length of hospital stay Number of raised ICP episodes per group Uncontrolled ICP, defined as instances of refractory ICP despite three consecutive doses of hyperosmolar therapy
Patil et al. [22]	Single-center 3-arm parallel RCT (India)	Adults 18 + years old with severe TBI due to road traffic accident and no immediate need for surgery GCS 8 or less, with sustained elevated ICP > 20 mm Hg for > 5 min *N* = 120	Variable bolus dose 3% hypertonic saline infused via CVC at infusion rate of 6 mL per minute until ICP < 15 mm Hg	Comparator 1: variable bolus dose 20% mannitol infused via CVC at infusion rate of 6 mL per minute Comparator 2: variable bolus dose 10% mannitol and 10% glycerol infused via CVC at infusion rate of 6 mL per minute Both comparators continued until ICP < 15 mm Hg	Reduction in ICP below 15 mm Hg (maximal ICP reduction)	None of interest
Roquilly et al. [11]	Multicenter RCT (France)	Adult (18–80 years old) patients with moderate to severe TBI defined as GCS 12 or lower and traumatic abnormal brain CT findings (e.g., extradural hematoma, subdural hematoma) Within 24 h of injury *N* = 370	Continuous intravenous infusion of 20% hypertonic saline at variable infusion rate for 48 h or longer if patients remained at high risk of raised ICP	Standard care to include hypertonic saline boluses, mannitol boluses, hypothermia, and other ICP-lowering methods	Extended GOS score at 6 months	Mortality rate in ICU Length of ICU stay Uncontrolled ICP, defined as ICP > 22 mm Hg for > 20 min Change in ICP during study period Hypernatremia
Vialet et al. [23]	Single-center parallel RCT (France)	Patients of all ages with head trauma and persistent coma and GCS less than 8 Requiring ICP monitoring and infusion of an osmotic agent to correct ICP *N* = 20	2-mL/kg bolus dose 7.5% hypertonic saline infused over 20 min Second dose given within 10 min if first dose failed	2-mL/kg bolus dose 20% mannitol infused over 20 min Second dose given within 10 min if first dose failed	ICP control, defined as the number of episodes and duration of intracranial hypertension per day	Uncontrolled ICP, defined as rate of failure of each treatment 90-day GOS score All-cause mortality by 6 months
Wahdan et al. [24]	Single-center parallel RCT (Egypt)	Adult patients aged 18–60 years with TBI GCS 4–12 *N* = 50	0.5-mL/kg/hour continuous hypertonic saline infusion over 48 h	3-mL/kg boluses (infused over 30 min) every 6 h for 48 h	None of interest	Length of ICU stay Mortality in ICU

*CT* computed tomography, *CVC* central venous catheter, *GCS* glasgow coma scale, *GOS* Glasgow Outcome Scale, *ICP* intracranial pressure, *ICU* intensive care unit, *RCT* randomized controlled trial, *TBI* traumatic brain injury

The majority of trials were conducted in patients aged 18 years and older. One trial included pediatric patients only (1–16 years old), whereas two others included patients aged 15–70 years and 16 years and older, respectively. One additional trial included patients of all ages. Six two-arm trials compared varying concentrations of intravenous (IV) HTS boluses with IV mannitol boluses. Two trials had three arms: one compared HTS boluses versus continuous HTS infusion versus mannitol boluses, and the other compared HTS boluses with two different concentrations of mannitol. One trial compared continuous HTS infusion with HTS boluses. Mannitol was the key comparator in the eight remaining trials. Concentrations and method of administration (bolus versus continuous infusion) of hyperosmolar agents varied between studies and are summarized in Table 1. One ongoing clinical trial was identified.

### Risk of Bias Assessment

**Fig. 3:**
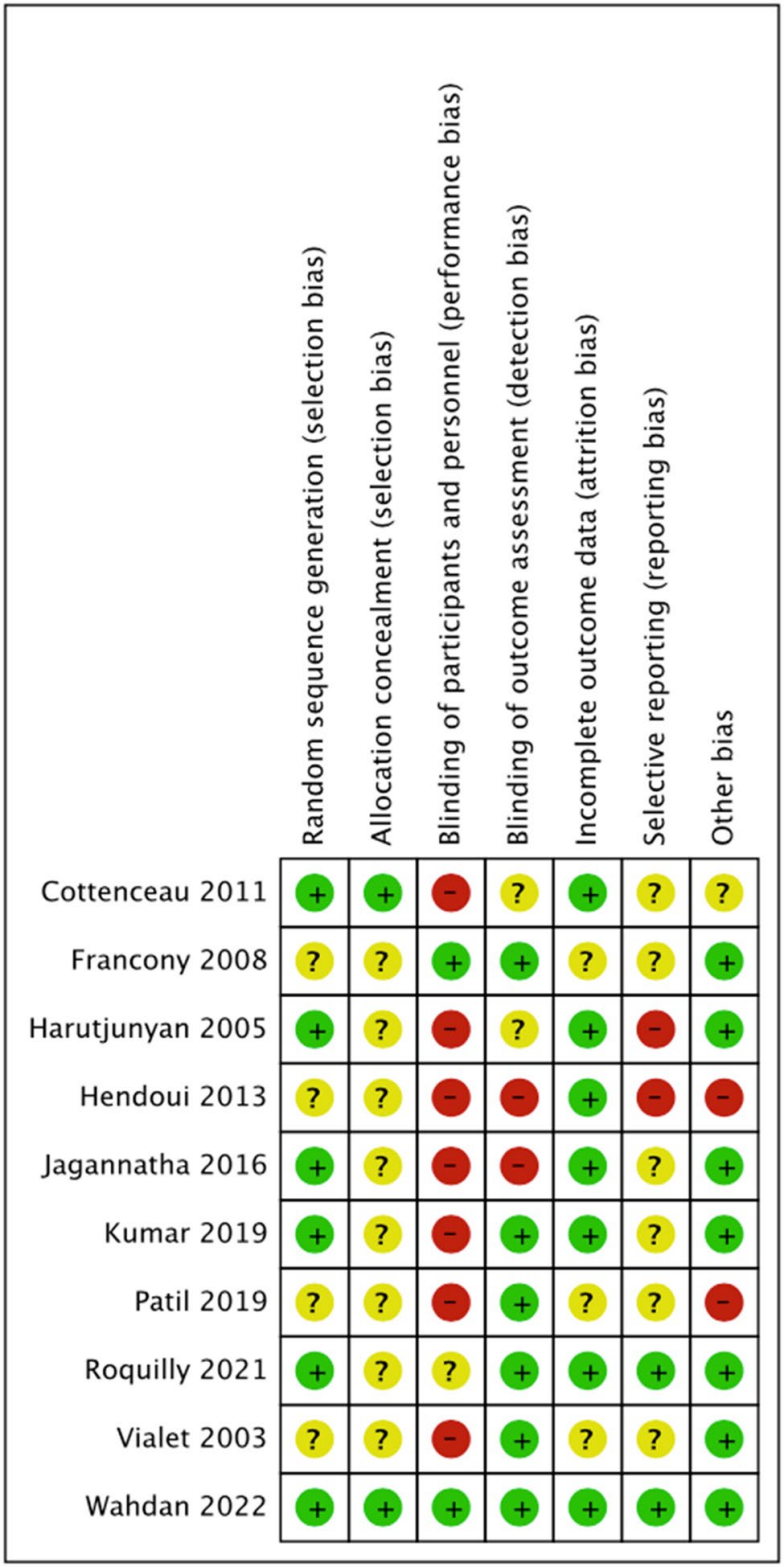
Risk of bias summary showing judgments about each risk of bias domain for each included study

The risk of bias assessment for individual trials is shown in Fig. 3. Nearly all trials were at high risk for lack of blinding of participants and personnel because of a presumed inability to blind interventions in the critical care setting. Two trials rated low risk for this domain prohibited any additional therapeutic intervention (for example, nursing, manipulation of ventilatory variables, or vasoactive support) during the study period. Allocation concealment was rated as unclear risk for eight trials because of lack of clarity in study methods. Similarly, protocols were unavailable for most included trials, which resulted in a rating of unclear risk of reporting bias for six trials. One trial was considered low risk for every domain [24].

### Effect of Interventions

#### GOS Score at 6 Months

**Fig. 4:**
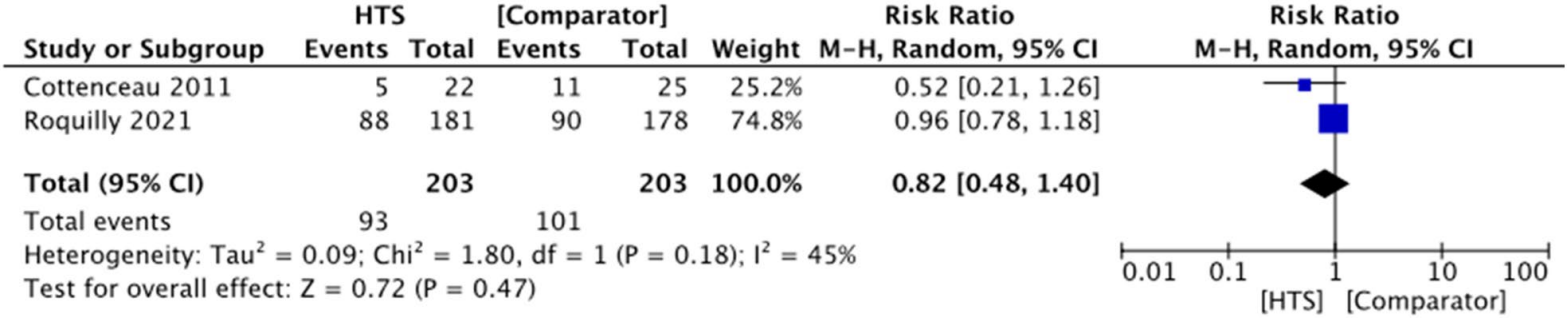
Forest plot showing effect of HTS versus comparator agents on favorable GOS score at 6 months

Five trials reported a GOS score at 6 months. However, only two of these trials were suitable for meta-analysis. There was no evidence of an effect of HTS on favorable GOS score in patients with acute TBI and raised ICP (RR 0.82, 95% CI 0.48–1.40, *P* = 0.47, *I*^2^ = 45%, 2 RCTs, 406 participants) (Fig. 4). The remaining three trials were reported narratively (Table 2) and showed no difference in GOS score between treatment groups (*P* > 0.05, 3 RCTs, *n* = 80) [20, 21, 23].

**Table 2:** Narrative synthesis for Glasgow Outcome Scale score

Study author	Number of patients	Comparison	Presentation of outcome	Results		Conclusions
				Intervention	Comparator	
Vialet et al. [23]	*N* = 20	7.5% HTS bolus versus 20% mannitol bolus	Reports number of patients with severe disability or deceased at 90 days	Severe disability, *n* = 6 Death, *n* = 4	Severe disability, *n* = 5 Death, *n* = 5	All patients in both groups either developed severe disability or died by 90 days. No meaningful difference in long-term outcome between groups
Kumar et al. [21]	*N* = 30	3% HTS bolus versus 20% mannitol bolus	Reports number of patients surviving with or without disability and number of patients in a vegetative state or deceased by 6 months	Survival with or without disability, *n* = 12	Survival with or without disability, *n* = 13	No meaningful difference in survival with or without disability
				Vegetative state or death, *n* = 2	Vegetative state or death, *n* = 3	No meaningful difference in rate of vegetative state or death
Jagannatha et al. [20]	*N* = 30	3% HTS bolus versus 20% mannitol bolus	Defines “favorable outcome” as “good recovery,” “moderate disability,” or “severe disability”	Favorable outcome, *n* = 2	Favorable outcome, *n* = 0	No meaningful difference in long-term outcome between treatment groups
			Defines “unfavorable outcome” as “persistent vegetative state” or “death”	Unfavorable outcome, *n* = 12	Unfavorable outcome, *n* = 16	

*HTS* hypertonic saline

#### All-Cause Mortality by 6 Months

**Fig. 5:**
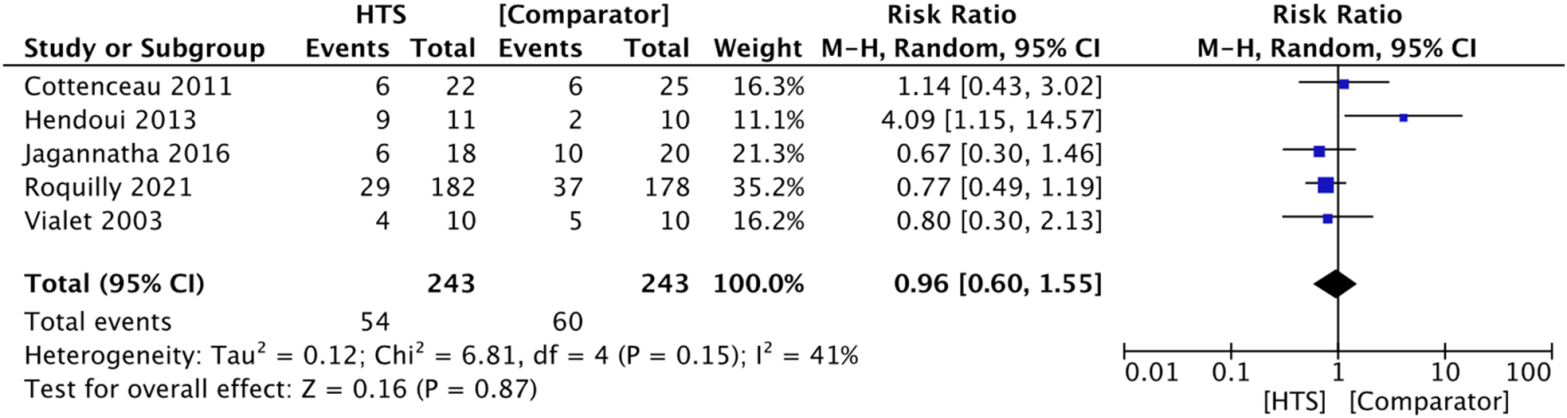
Forest plot showing effect of HTS versus comparator agents on all-cause mortality by 6 months

There was no evidence of an effect of HTS on all-cause mortality by 6 months in patients with acute TBI (RR 0.96, 95% CI 0.60–1.55, *P* = 0.87, *I*^2^ = 41%, 5 RCTs, 486 participants) (Fig. 5). An additional trial comparing continuous 3% HTS infusion with intermittent 3% HTS boluses reported no difference in ICU mortality between the two groups, suggesting that the mode of HTS delivery had no impact on early mortality (*P* > 0.05, 50 participants) [24]. No trials reported reasons for deaths, but results from the COBI trial suggest that nearly all deaths occurred in both groups within the first 100 days from randomization [11].

#### Adverse Hypernatremia

**Fig. 6:**
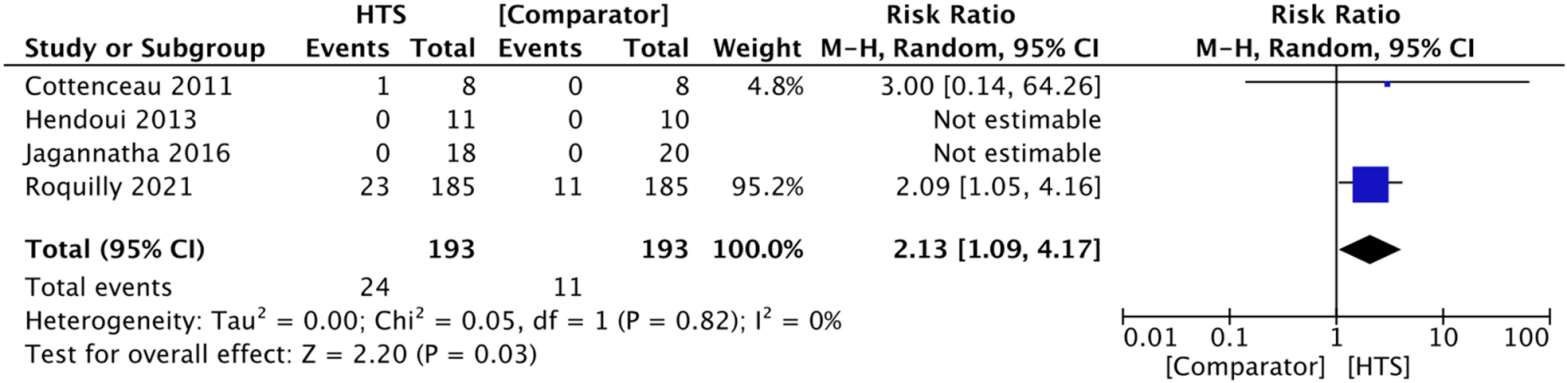
Forest plot showing effect of HTS versus comparator agents on the rate of adverse hypernatremia

There was variation in study authors’ definitions of adverse hypernatremia across the four trials included (Table 3). Two trials reported zero events in both the HTS and comparison groups [19, 20]. Therefore, only two studies were included in the meta-analysis, which showed that HTS use is associated with an increased risk of hypernatremia (RR 2.13, 95% CI 1.09–4.17, *P* = 0.03, *I*^2^ = 0%, 2 RCTs, 386 participants) (Fig. 6) [11, 16]. However, it should be noted that the multicenter COBI RCT comparing continuous infusion of a high concentration of HTS (20%) with other ICP-lowering agents accounted for the majority of the weighting for this point estimate [11]. Thus, it is possible that the reason for a higher risk of severe hypernatremia in the intervention group is largely due to the high concentration of HTS given continuously for at least 48 h, and these results should be interpreted within this context.

**Table 3:** Definitions of “adverse hypernatremia” for each study

Study author	Definition of “adverse hypernatremia”
Cottenceau et al. [16]	Defined as plasma sodium level high enough to necessitate cessation of HTS infusion
Hendoui et al. [19]	Defined as plasma sodium concentration greater than 155 mEq/L
Jagannatha et al. [20]	Defined as plasma sodium concentration greater than 160 mM
Roquilly et al. [11]	Defined as plasma sodium level greater than 160 mM

*HTS* hypertonic saline

#### Uncontrolled ICP

**Fig. 7:**
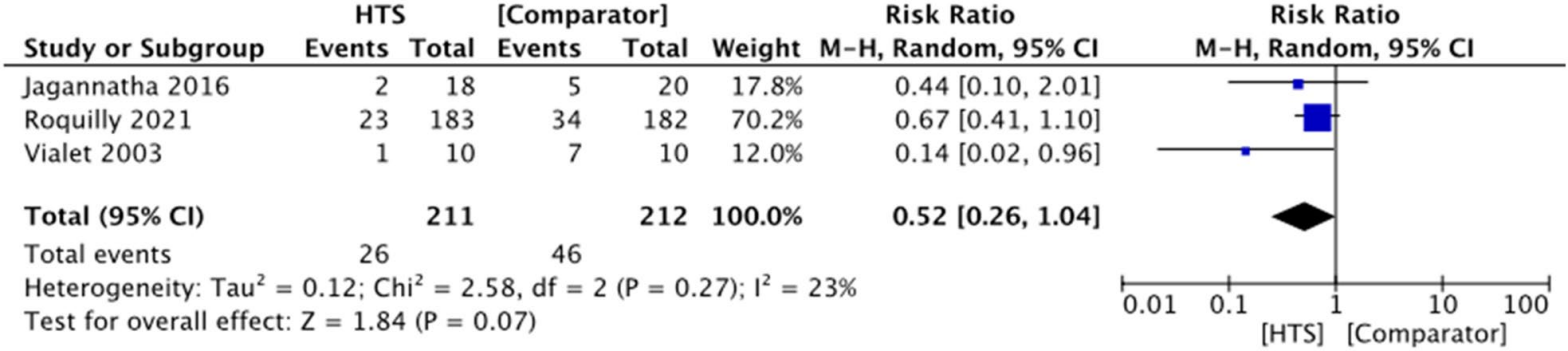
Forest plot showing effect of HTS versus comparator agents on the rate of uncontrolled ICP after intervention

Six trials reported “uncontrolled ICP” as an outcome, of which three were included in the meta-analysis [11, 20, 23]. Definitions of this outcome varied. One study defined this outcome as requirement of Brain Trauma Foundation guidelines “stage 3 therapies,” including barbiturates to lower ICP [11]. Vialet et al. [23] defined treatment failure as sustained raised ICP greater than 35 mm Hg despite two consecutive infusions of hyperosmolar therapy. Finally, Jagannatha et al. [20] defined this outcome as “persistently elevated ICP greater than 20 mmHg despite a maximum of three doses of hyperosmolar therapy,” necessitating the use of further ICP-lowering measures, including barbiturates, propofol, hyperventilation, cerebral spinal fluid drainage, or decompressive craniectomy. The meta-analysis showed no evidence of an effect of HTS on reducing ICP compared with other agents (RR 0.52, 95% CI 0.26–1.04, *P* = 0.07, *I*^2^ = 23%, 3 RCTs, 423 participants) (Fig. 7).

Three trials reported this outcome as follows: average time ICP exceeded 20 mm Hg, barbiturate requirement, and/or episodes of refractory ICP after three consecutive doses of hyperosmolar therapy [16, 21, 23]. Collectively, results from two of the trials showed no difference in the incidence of uncontrolled ICP between HTS and comparator groups (*P* > 0.05, 3 RCTs, 62 participants) [16, 21]. One trial showed that ICP exceeded 25 mm Hg for a shorter duration of time in the HTS group compared with the control group, although the authors did not provide baseline ICP data from participants at the start of the study period, which complicates interpretation of this result [23].

#### Length of stay (hospital or ICU)

**Fig. 8:**
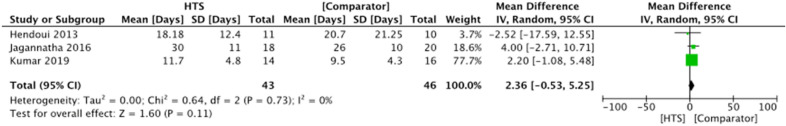
Forest plot showing effect of HTS versus comparator agents on total length of hospital stay

**Fig. 9:**
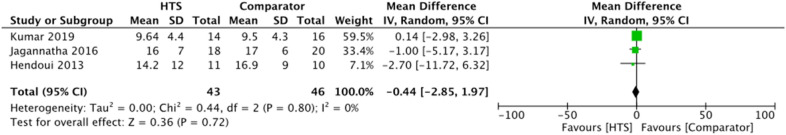
Forest plot showing effect of HTS versus comparator agents on length of ICU stay

The meta-analysis showed no evidence of an effect of HTS on total length of hospital stay compared with comparator agents (RR 2.36, 95% CI − 0.53 to 5.25, *P* = 0.11, *I*^2^ = 0%, 3 RCTs, 101 participants) (Fig. 8). Similarly, the meta-analysis showed no evidence of an effect of HTS on length of ICU stay (RR − 0.44, 95% CI − 2.85 to 1.97, *P* = 0.72, *I*^2^ = 0%, 3 RCTs, 101 participants) (Fig. 9). One additional trial reported no difference in length of ICU stay as a median and interquartile range (median 16 days in HTS group compared with 15 days in control group, difference = 1.0 day, 95% CI − 1.0 to 4.0 days, 370 participants) [11]. Additionally, Wahdan et al. [24] reported no difference in length of ICU stay when comparing continuous 3% HTS infusion with intermittent 3% HTS boluses (17.5 ± 11.8 and 17.2 ± 12.9, respectively, *P* = 0.36, 50 participants).

#### Reduction in ICP

Five trials reported ICP reduction as an outcome (605 participants), and these are described in Table 4 [11, 16, 20–22]. These trials could not be included in the meta-analysis because of variability of outcome reporting. Overall, there was no consistent effect of HTS on lowering ICP compared with other agents in patients with acute TBI.

**Table 4:** Narrative synthesis for intracranial pressure reduction

Study author	*N*	Comparison	Definition of outcome(s)	Results		Conclusions
				Intervention	Comparator(s)	
Cottenceau et al. [16]	*N* = number of boluses given over 3 days 47 patients	7.5% HTS bolus versus 20% mannitol bolus	Mean absolute ICP reduction at 30 min after bolus administration, mean (SEM)	7 (2.0)	9 (1.0)	No significant difference in ICP reduction between groups (*P* > 0.05)
Jagannatha et al. [20]	*N* = number of boluses given for each ICH episode 38 patients	3% HTS bolus versus 20% mannitol bolus	Mean ICP reduction (mm Hg), mean (SD)	10.1 (8.7)	8.9 (8.4)	No significant difference in ICP reduction between groups (*P* = 0.135)
			Mean duration of ICP reduction (minutes), mean (SD)	55 (32)	57 (31)	No significant difference in duration of ICP reduction between groups (*P* = 0.4)
Kumar et al. [21]	*N* = number of patients 30 patients	3% HTS bolus versus 20% mannitol bolus	Mean ICP reduction, mean (SD)	5.67 (3.9)	7.13 (2.9)	No significant difference in ICP reduction between groups (*P* = 0.33)
Patil et al. [22]	*N* is unclear from study results 120 patients	3% HTS bolus versus 20% mannitol versus 10% mannitol + 10% glycerol	Percentage reduction (%) in ICP calculated as follows: (Max. ICP – Min. ICP)/Max. ICP	− 60%	20% mannitol: − 55% 10% mannitol + 10% glycerol: − 57%	No significant difference in percentage ICP decrease between groups (*P* > 0.05)
Roquilly et al. [11]	*N* = number of patients 370 patients	Continuous 20% HTS infusion versus standard care alone	Mean difference in ICP reduction (mmHg)	Authors do not provide raw data for each group, but do provide a mean difference (95% CI) of − 1.29 (− 2.89 to 0.3)		There was no significant difference in ICP between groups

*CI* confidence interval, *HTS* hypertonic saline, *ICH* intracranial hemorrhage, *ICP *intracranial pressure, *Max*. maximum, *Min*. minimum

#### Pulmonary Edema and Rebound Phenomenon

Although pulmonary edema and rebound phenomenon are potential complications of HTS use [16, 17], none of the included trials reported either as outcomes, with the exception of Francony et al. [17], who reported that there were no instances of rebound phenomenon during the study.

### Certainty of Evidence

The certainty of evidence ranged from low to very low across all outcomes (Table 5). Common reasons for downgrading were imprecision, differences in estimated effect size, and suspected publication bias.

**Table 5:** Summary of findings: HTS compared with control for patients with acute traumatic brain injury

Outcomes	Anticipated absolute effects^a^ (95% CI)		Relative effect (95% CI)	Number of participants (studies)	Certainty of the evidence (GRADE)	Comments
	Risk with control	Risk with HTS				
All-cause mortality by 6 months	247 per 1000	237 per 1000 (148 to 383)	RR 0.96 (0.60–1.55)	486 (5 RCTs)	⨁⨁◯◯ Low^b^^,c^	The evidence suggests that HTS results in little to no difference in all-cause mortality by 6 months
Hypernatremia	57 per 1000	121 per 1000 (62 to 238)	RR 2.13 (1.09–4.17)	386 (2 RCTs)	⨁⨁◯◯ Low^d^^,e^	HTS results in an increase in hypernatremia
Uncontrolled intracranial pressure	217 per 1000	113 per 1000 (56 to 226)	RR 0.52 (0.26–1.04)	423 (3 RCTs)	⨁⨁◯◯ Low^f^^,g^	The evidence suggests that HTS results in little to no difference in uncontrolled intracranial pressure
Total length of hospital stay (days)	–	MD 2.36 higher (0.53 lower to 5.25 higher)	–	89 (3 RCTs)	⨁◯◯◯ Very low^h^^,i,j^	The evidence is very uncertain about the effect of HTS on length of hospital stay (days)
Length of ICU stay (days)	–	MD 0.44 lower (2.85 lower to 1.97 higher)	–	89 (3 RCTs)	⨁◯◯◯ Very low^h,i,j^	The evidence is very uncertain about the effect of HTS on length of ICU stay (days)
Favorable Glasgow Outcome Scale Score at 6 months	498 per 1000	408 per 1000 (239 to 697)	RR 0.82 (0.48–1.40)	406 (2 RCTs)	⨁⨁◯◯ Low^k,l,m,n^	HTS probably results in little to no difference in favorable Glasgow Outcome Scale Score

Patient or population: patients of all ages with acute traumatic brain injury; setting: critical care; intervention: HTS; comparison: comparator ICP-lowering agents

*CI* confidence interval, *GRADE* Grading of Recommendations Assessment, Development, and Evaluation, *HTS* hypertonic saline, *ICP* intracranial pressure, *ICU* intensive care unit, *MD* mean difference, *RCT* randomized controlled trial, *RR* risk ratio

^a^The risk in the intervention group (and its 95% CI) is based on the assumed risk in the comparison group and the relative effect of the intervention (and its 95% CI)

^b^CIs for most included studies are wide. Four of five included studies have small sample sizes with a small combined event number

^c^Funnel plot asymmetrical

^d^CIs are reasonably wide for both studies included. One study has a very small sample size (Cottenceau et al. [16]) with a very low event rate

^e^Funnel plot asymmetrical. Few studies

^f^Jagannatha et al. [20] and Vialet et al. [23] have small sample sizes and event rates (wide CIs as a result)

^g^Funnel plot asymmetrical. Few studies

^h^CIs overlap, *P* > 0.05 (heterogeneity), I^2^ = 0%. There is considerable difference in effect size, but this may be attributable to the fact that the study by Kumar et al. [21] is a pediatric study (ages 1–16) and accounts for the majority of the weighting for the point estimate (77.1%). The study by Jagannatha et al. [20] includes ages 15–70 years, and the study by Hendoui et al. [19] includes adult patients only aged 18–65 years. Thus, there are considerable differences in baseline characteristics (particularly age) between studies, which may account for the differences in effect sizes

^i^All included studies have small sample sizes and wide CIs

^j^Funnel plot asymmetrical. Few studies

^k^CIs overlap, *P* > 0.05 (heterogeneity), I^2^ = 45% (low-moderate), reasonable difference in effect size (0.52 vs. 0.96), but this could be explained by the difference in HTS administration methods between studies (continuous HTS infusion versus HTS boluses as needed)

^l^Data provided by two trials. The study by Roquilly et al. [11] accounts for the majority of weighting toward the point estimate (74.8%). There are important differences in patient populations between studies. For example, the study by Cottenceau et al. [16] includes patients aged 16 + years of age and only patients with severe traumatic brain injury. The study by Roquilly et al. [11] includes adult patients of 18–80 years of age with moderate to severe traumatic brain injury (glasgow coma scale 12 or lower), which introduces a range of traumatic brain injury severity to the patient population. Moreover, in the study by Roquilly et al. [11], 20% HTS is delivered via continuous infusion (over 48 + hours). In the study by Cottenceau et al. [16], 7.5% HTS (much lower concentration) is delivered via bolus over 20 min

^m^95% CI fairly wide

^n^Funnel plot asymmetrical. Few studies

## Discussion

### Key Findings

Our systematic review identified ten RCTs enrolling 760 patients of all ages with acute TBI. The main findings were the following: (1) there was no evidence of an effect of HTS compared with other agents (mainly mannitol) on long-term neurological outcome in patients with raised ICP; (2) similarly, there was no evidence of a beneficial effect of HTS on all-cause mortality, uncontrolled ICP, length of hospital or ICU stay, and ICP reduction; and (3) HTS may be associated with increased risk of adverse hypernatremia. However, 95% CIs were wide for all studied outcomes. Thus, it is difficult to elucidate clinically meaningful differences between HTS and other ICP-lowering strategies, including mannitol.

Overall, our results challenge previous studies [6, 7] that suggest HTS is more effective than its comparators (e.g., mannitol) and are congruent with findings of a recent Cochrane review that showed there is weak evidence that HTS is no better than mannitol for long-term management of TBI [10]. Despite the finding that HTS is associated with adverse hypernatremia compared with other agents, this result should be interpreted with caution because one large multicenter trial accounts for the majority of the weighting for this point estimate [11]. Importantly, this trial investigated the continuous infusion (at least 48 h) of a higher concentration of HTS (20%) than is normally used clinically (range 1.8–5%). Thus, it is possible that prolonged continuous infusion of concentrated HTS is largely responsible for the apparent increased risk of hypernatremia in the patients studied. On the contrary, other studies have reported no difference in plasma sodium concentration when comparing patients receiving HTS versus those receiving mannitol boluses, which might suggest a failure to achieve a hyperosmolar state when certain administration techniques are used [17, 20]. The effect of bolus versus continuous infusion of HTS on plasma sodium levels should be explored further to determine whether there is an optimum administration method to achieve a therapeutic hyperosmolar state without resulting in adverse hypernatremia.

### Implications for Practice

Despite a lack of clarity regarding the benefits of HTS in the management of acute TBI, a recent practice survey reported that most UK centers are moving to the use of HTS as first-line hyperosmolar therapy over mannitol [25, 26]. Use of near-patient sodium monitoring (e.g., blood gas analysis) may, in part, make it easier for clinicians to use and titrate HTS. This review shows that there is currently insufficient evidence to make a recommendation for HTS over other ICP-lowering agents in patients with acute TBI. However, it should be noted that this evidence is of low or very low certainty, and any beneficial effect of HTS would need to be balanced against the potential risk of hypernatremia.

### Implications for Research

Currently, there is a paucity of large-scale RCT data comparing ICP-lowering agents in the context of TBI. This is partly explained by the relatively rare prevalence of severe TBI necessitating the use of ICP-lowering agents in critical care settings, which imposes limits on trial recruitment. Similarly, there is a lack of available RCT data from lower middle-income countries and pediatric populations. For instance, this review includes only one pediatric study reporting a GOS score [21]. This limits the generalizability of the findings in this review, which includes trials enrolling predominantly adult patients from higher-income countries. There is a need for larger international and multicenter trials in a variety of settings to address the current lack of high-quality evidence and to determine whether there are preferred ICP-lowering therapies in specific patient populations. The ongoing Sugar or Salt phase III trial (including 25–28 ICUs across the United Kingdom) may provide further clarity on benefits or risks associated with the use of HTS in patients with acute TBI (ISRCTN16075091) [26].

Finally, heterogeneous reporting of outcomes after TBI (including long-term functional outcome scores such as the GOS score) across clinical trials compromises the validity of comparison between studies and hinders progress in this field. This review highlights the inconsistency in TBI outcome reporting. For instance, three trials included in narrative syntheses for this review reported GOS scores in forms that were not amenable to inclusion in a pooled analysis. Vialet et al. [23] only reported the number of patients with severe disability or who were deceased at 90 days. Jagannatha et al. [20] defined “favorable” outcome as “good recovery,” “moderate disability,” or “severe disability,” which is likely to be at odds with what most patients would consider to be favorable. Furthermore, Kumar et al. [21] reported the number of patients surviving with or without disability and the number of patients in a vegetative state or deceased by 6 months [21]. These methods of GOS reporting are unlikely to be helpful to clinicians or patients and emphasize the need for a standardized core outcome set for TBI. The core outcome set for trials in significant traumatic brain injury (COSTS-TBI) project aimed to develop a core outcome set to set a standard for future trials including patients with moderate to severe TBI but has since been withdrawn in 2021 [27]. Working toward an international consensus on TBI outcome reporting standards will enable meaningful comparison of trial data worldwide and will allow for better assessment of ICP-lowering therapies in different critical care settings. Further consensus on thresholds for adverse hypernatremia and optimum monitoring of plasma sodium concentration and clinical features in patients receiving HTS will be helpful in the assessment of this outcome.

### Strengths and limitations

This review followed a strict methodological process, adhering to Cochrane, PRISMA, and GRADE recommendations. We have also included recently published data from the COBI trial [11], which is the largest RCT investigating the use of HTS for acute TBI to date and has been excluded from previous reviews on this subject [10, 28]. Limitations of this review can be attributed to the clinical and methodological differences between trials, which also included generally small sample sizes. Moreover, differences in outcome reporting methods limited the data suitable for inclusion in meta-analyses and precluded sensitivity and subgroup analyses based on age group, TBI severity, and administration methods. As a result, it is still unclear whether there is an optimum hyperosmolar therapy depending on patient age group or severity of TBI. Additionally, dichotomization of the primary outcome into “favorable” versus “unfavorable” outcomes required us to make judgments about what most patients and clinicians would consider to be a reasonable dichotomy. This was considered necessary to enable meta-analysis because of the variation in GOS reporting across the included trials, and some provided data for pooled GOS scores rather than for each individual GOS score. Thus, it is possible that important information about long-term neurological outcome that could influence or guide patient and clinician decisions is not represented in these findings.

## Conclusions

Despite increased popularity in its use, we have shown that there is no evidence of an effect of intravenous HTS compared with other ICP-lowering hyperosmolar agents (mannitol) on important outcomes of interest, including long-term neurological function (measured by GOS score), all-cause mortality, uncontrolled ICP, and length of hospital or ICU stay. HTS may be associated with higher risk of hypernatremia. However, this conclusion is based on very low to low certainty evidence, and clinicians must balance any benefits of HTS with the risk of hypernatremia. In the future, larger well-designed trials investigating the use of hyperosmolar agents in patients with TBI with a comprehensive core outcome set are required to provide further clarity and to guide clinical practice. Overall, these results do not support a recommendation for use of HTS over mannitol in treatment of patients with raised ICP secondary to acute TBI.

### Supplementary Information

Below is the link to the electronic supplementary material.

Supplementary file1 (DOCX 319 kb)

Supplementary file2 (DOCX 30 kb)
