# Supplementary material for: Hypertonic Saline Versus Other Intracranial-Pressure-Lowering Agents for Patients with Acute Traumatic Brain Injury: A Systematic Review and Meta-analysis
Source: Neurocrit Care. 2023 Jun 28;40(2):769–84. doi: 10.1007/s12028-023-01771-9 (PMC10959781; doi:10.1007/s12028-023-01771-9)
Supplement: Supplementary file 1 — Supplementary file1 (DOCX 319 kb) [file 12028_2023_1771_MOESM1_ESM.docx]

**Appendices**

**Appendix 1: Completed PRISMA checklist**

(separate document)

**Appendix 2: Search strategies**

**Ovid MEDLINE**

| Search line | Search term(s) | Results |
| --- | --- | --- |
| 1 | *Saline Solution, Hypertonic/ | 2843 |
| 2 | infusion.mp. [mp=title, abstract, original title, name of substance word, subject heading word, floating sub-heading word, keyword heading word, organism supplementary concept word, protocol supplementary concept word, rare disease supplementary concept word, unique identifier, synonyms] | 239942 |
| 3 | 1 and 2 | 689 |
| 4 | (hypertonic adj3 saline adj3 infusion).mp. [mp=title, abstract, original title, name of substance word, subject heading word, floating sub-heading word, keyword heading word, organism supplementary concept word, protocol supplementary concept word, rare disease supplementary concept word, unique identifier, synonyms] | 906 |
| 5 | 3 or 4 | 1338 |
| 6 | ((traumatic adj2 (brain or cerebr*) adj2 injur*) or TBI).mp. [mp=title, abstract, original title, name of substance word, subject heading word, floating sub-heading word, keyword heading word, organism supplementary concept word, protocol supplementary concept word, rare disease supplementary concept word, unique identifier, synonyms] | 49764 |
| 7 | Brain Injuries, Traumatic/ | 10207 |
| 8 | 6 or 7 | 49764 |
| 9 | 5 and 8 | 50 |
| 10 | (intracranial pressure or ICP).mp. [mp=title, abstract, original title, name of substance word, subject heading word, floating sub-heading word, keyword heading word, organism supplementary concept word, protocol supplementary concept word, rare disease supplementary concept word, unique identifier, synonyms] | 48776 |
| 11 | (intracranial adj2 hypertension).mp. [mp=title, abstract, original title, name of substance word, subject heading word, floating sub-heading word, keyword heading word, organism supplementary concept word, protocol supplementary concept word, rare disease supplementary concept word, unique identifier, synonyms] | 11825 |
| 12 | 10 or 11 | 54793 |
| 13 | 9 and 12 | 30 |
| 14 | limit 13 to yr="2000 -Current" | 29 |
| 15 | limit 14 to randomized controlled trial | 5 |

**Embase**

| ch line | Search term(s) | Results |
| --- | --- | --- |
| 1 | *sodium chloride/ | 32574 |
| 2 | hypertonic.mp. [mp=title, abstract, heading word, drug trade name, original title, device manufacturer, drug manufacturer, device trade name, keyword heading word, floating subheading word, candidate term word] | 20141 |
| 3 | infusion.mp. [mp=title, abstract, heading word, drug trade name, original title, device manufacturer, drug manufacturer, device trade name, keyword heading word, floating subheading word, candidate term word] | 401846 |
| 4 | 1 and 2 and 3 | 981 |
| 5 | (hypertonic adj3 saline adj3 infusion).mp. [mp=title, abstract, heading word, drug trade name, original title, device manufacturer, drug manufacturer, device trade name, keyword heading word, floating subheading word, candidate term word] | 1018 |
| 6 | 4 or 5 | 1676 |
| 7 | ((traumatic adj2 (brain or cerebr*) adj2 injur*) or TBI).mp. [mp=title, abstract, heading word, drug trade name, original title, device manufacturer, drug manufacturer, device trade name, keyword heading word, floating subheading word, candidate term word] | 87522 |
| 8 | exp traumatic brain injury/ | 58338 |
| 9 | 7 or 8 | 87982 |
| 10 | 6 and 9 | 113 |
| 11 | (intracranial pressure or ICP).mp. [mp=title, abstract, heading word, drug trade name, original title, device manufacturer, drug manufacturer, device trade name, keyword heading word, floating subheading word, candidate term word] | 64765 |
| 12 | intracranial hypertension.mp. [mp=title, abstract, heading word, drug trade name, original title, device manufacturer, drug manufacturer, device trade name, keyword heading word, floating subheading word, candidate term word] | 23848 |
| 13 | 11 or 12 | 77663 |
| 14 | 10 and 13 | 85 |
| 15 | limit 14 to yr="2000 -Current" | 83 |
| 16 | limit 15 to randomized controlled trial | 9 |

**Cochrane CENTRAL advanced search**

(hypertonic AND saline AND infusion) OR (HTS infusion) in Title Abstract Keyword AND "traumatic brain injury" OR TBI OR traumatic brain injur* in Title Abstract Keyword AND "randomised controlled trials" OR RCT OR randomis* control* trial* in All Text NOT sodium lactate in Title Abstract Keyword AND intracranial pressure OR ICP OR intracranial hypertension in Title Abstract Keyword - (Word variations have been searched)

Filtered by 2000-2022

12 results

**SCOPUS**

((TITLE-ABS-KEY (hypertonic AND saline) AND TITLE-ABS-KEY (traumatic AND brain AND injury OR tbi) AND TITLE-ABS-KEY ((intracranial AND pressure) OR icp OR (intracranial AND hypertension)) AND NOT TITLE-ABS-KEY ((sodium AND lactate))) AND PUBYEAR > 1999 ) AND (rct OR randomised AND control AND trial) AND (LIMIT-TO (DOCTYPE, “ar”))

25 results


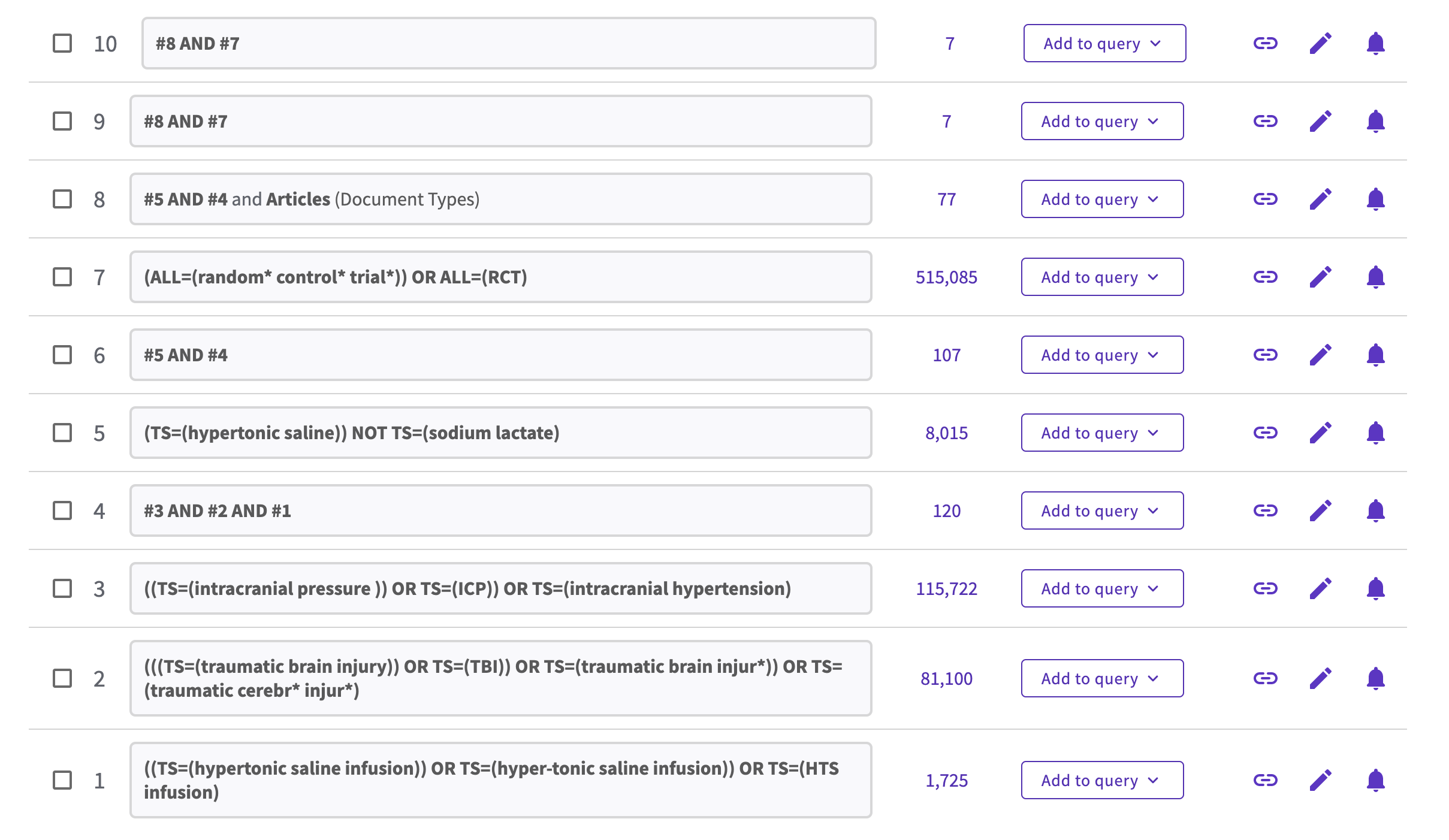
**Web of Science**

7 results

**Appendix 3: Glasgow Outcome Scale score criteria**

*5-point scale:*

1. Good Recovery
2. Moderate Disability
3. Severe Disability
4. Survival with vegetative state
5. Death

*Extended GOS (8-point scale):*

1. Upper good recovery
2. Lower good recovery
3. Upper moderate disability
4. Lower moderate disability
5. Upper severe disability
6. Lower severe disability
7. Vegetative state
8. Death
